# Supplementary material for: ProteinShader: illustrative rendering of macromolecules
Source: BMC Struct Biol. 2009 Mar 30;9:19. doi: 10.1186/1472-6807-9-19 (PMC2672931; doi:10.1186/1472-6807-9-19)
Supplement: Additional file 1 — ProteinShader program without source code. This compressed file contains the complete ProteinShader program including associated libraries, but no source code. A README.txt file gives an overview of the ProteinShader distribution, and the index.html file in the help subdirectory has directions on getting started with the program as well as a set of tutorials. [file 1472-6807-9-19-S1.zip › ProteinShader-beta-0_9_4-binary/help/api/org/proteinshader/structure/class-use/BetaStrand.html]

Uses of Class org.proteinshader.structure.BetaStrand (ProteinShader API)


|  |  |  |
| --- | --- | --- |
| **Overview** \| **Package** \| **Class** \| **Use** \| **Tree** \| **Deprecated** \| **Index** \| **Help** | |  |
| PREV   NEXT | **FRAMES**    **NO FRAMES**     **All Classes** |  |


---


## **Uses of Class org.proteinshader.structure.BetaStrand**

| Packages that use BetaStrand | |
| --- | --- |
| **org.proteinshader.structure** | Holds the classes that store information from a Protein Data Bank file: Structure, Model, Chain, AminoAcid, Heterogen, Water, Atom, Bond, Helix, BetaStrand, Loop, *etc*. |
| **org.proteinshader.structure.visitor** | Holds Visitor classes that know how to traverse the hierarchy of objects held by class Structure. |

Uses of BetaStrand in org.proteinshader.structure

| Methods in org.proteinshader.structure that return BetaStrand | |
| --- | --- |
| `BetaStrand` | `Chain.addNewBetaStrand(String betaStrandID, String betaStrandNumber, String sheetID, String startResidueID, String endResidueID, int sense, int strandsInSheet)`             Creates a new BetaStrand with the BetaStrand ID given as an argument, adds the BetaStrand to the Chain's collection of BetaStrands, and returns a reference to the new BetaStrand. |
| `BetaStrand` | `Chain.getBetaStrand(String betaStrandID)`             Returns the BetaStrand with the betaStrandID given as an argument. |
| `BetaStrand` | `Model.getBetaStrand(String betaStrandID)`             Returns the BetaStrand with the betaStrandID given as an argument. |
| `BetaStrand` | `Structure.getBetaStrand(String betaStrandID, String modelID)`             Returns the BetaStrand with the IDs given as arguments. |

| Methods in org.proteinshader.structure that return types with arguments of type BetaStrand | |
| --- | --- |
| `Iterator<BetaStrand>` | `Chain.iteratorBetaStrands()`             Returns an Iterator for the BetaStrands held by this Chain. |
| `Iterator<BetaStrand>` | `Model.iteratorBetaStrands()`             Returns an Iterator for the BetaStrands held by this Model. |

Uses of BetaStrand in org.proteinshader.structure.visitor

| Methods in org.proteinshader.structure.visitor with parameters of type BetaStrand | |
| --- | --- |
| `void` | `Visitor.visit(BetaStrand betaStrand)`             If debug is true, the betaStrandID, startResidueID, and endResidueID will be printed to standard out. |
| `void` | `SFWriterVisitor.visit(BetaStrand betaStrand)`             Writes all information held by a BetaStrand. |

---


|  |  |  |
| --- | --- | --- |
| **Overview** \| **Package** \| **Class** \| **Use** \| **Tree** \| **Deprecated** \| **Index** \| **Help** | |  |
| PREV   NEXT | **FRAMES**    **NO FRAMES**     **All Classes** |  |


---

# *Copyright © 2007-2008*
